# The Number of Cultural Traits Is Correlated with Female Group Size but Not with Male Group Size in Chimpanzee Communities

**DOI:** 10.1371/journal.pone.0009241

**Published:** 2010-03-24

**Authors:** Johan Lind, Patrik Lindenfors

**Affiliations:** Centre for the Study of Cultural Evolution & Department of Zoology, Stockholm University, Stockholm, Sweden; University of Oxford, United Kingdom

## Abstract

What determines the number of cultural traits present in chimpanzee (*Pan troglodytes*) communities is poorly understood. In humans, theoretical models suggest that the frequency of cultural traits can be predicted by population size. In chimpanzees, however, females seem to have a particularly important role as cultural carriers. Female chimpanzees use tools more frequently than males. They also spend more time with their young, skewing the infants' potential for social learning towards their mothers. In Gombe, termite fishing has been shown to be transmitted from mother to offspring. Lastly, it is female chimpanzees that transfer between communities and thus have the possibility of bringing in novel cultural traits from other communities. From these observations we predicted that females are more important cultural carriers than males. Here we show that the reported number of cultural traits in chimpanzee communities correlates with the number of females in chimpanzee communities, but not with the number of males. Hence, our results suggest that females are the carriers of chimpanzee culture.

## Introduction

Chimpanzee (*Pan troglodytes*) cultures exhibit considerable variation between communities [1]. Some of the variation in culture among apes can be explained by local ecological conditions and the diffusion and differentiation of cultural traits between communities [2]–[5]. We here want to propose another important correlate of chimpanzee culture: female group size.

Cultural traits are carried by individuals and inherited through social learning. Thus, the number of cultural traits that can exist in a population depends on the number of individuals that are available to learn from. The diversity of cultural traits present in human populations can be theoretically predicted to increase with community size [6]–[7]. This relationship potentially explains the geographic variation in the timing of the first appearance of modern behaviour, as manifested through advanced human culture, during the Pleistocene without invoking increased cognitive capacity [7]. Here we test if the relationship between cultural diversity and community size holds true also for chimpanzees.

However, there are four key reasons for why adult females can be suspected to be of particular importance for cultural transmission in chimpanzees. First, it can be predicted that young chimpanzees learn more from their mothers than from any other individual in the community since young chimpanzees depend on their mothers up to eight years whereas male involvement is scarce [8]–[9]. Second, tool use is central in chimpanzee culture and females use tools more frequently than males [8], [10]–[11]. Hence, an important part of chimpanzee culture is mainly exhibited by females. Third, in a detailed study of how chimpanzees in Gombe learn to fish for termites it was found that the time the mother spent termite-fishing was positively correlated to the offspring's acquisition of critical elements of the skill [12]. Since mothers spend more time with their offspring in general, the same pattern can reasonably be expected also for other traits. Fourth, it is the females that transfer between communities in chimpanzees, not the males [9]. Thus, traits learnt by males stay within the community, while traits learnt by females can be transferred to other communities. Even if only a sub-section of females' cultural repertoires are unique to each particular female, the diversity of cultural traits can be predicted to be larger among females than among males.

Because females express and transmit more culture than males, and because females transfer between communities bringing with them their cultural knowledge, the number of cultural traits present in any given chimpanzee community should depend on the number of females in that community. Thus, we hypothesize that the number of cultural traits in chimpanzee communities should correlate with the average number of females in chimpanzee communities, but not with the average number of males.

## Results

Since variation in research effort potentially can bias the diversity of cultural traits reported in different communities, we tested if the length of each long-term project affected the reported number of cultural traits (we used the following start dates for the six different projects: Bossou: 1976, Taï: 1979, Gombe: 1960, Mahale: 1965, Kibale: 1987, Budongo: 1990). There was no correlation between the length of the studies and the number of reported cultural traits (r_s_ = 0.450, p = 0.312, n = 6) so we suspect no such bias.

To ascertain the independence of the cultural and community data we estimated λ, a statistic that varies between 0 (phylogenetic independence) and 1 (species' traits covary in direct proportion to their shared evolutionary history) [13]. This parameter did not differ significantly from 0.0 in any test (p>0.4 for all parameters) indicating no presence of a phylogenetic signal in our data (but see 5). Henceforth we therefore only report the results of non-phylogenetic tests.

**Figure 1:**
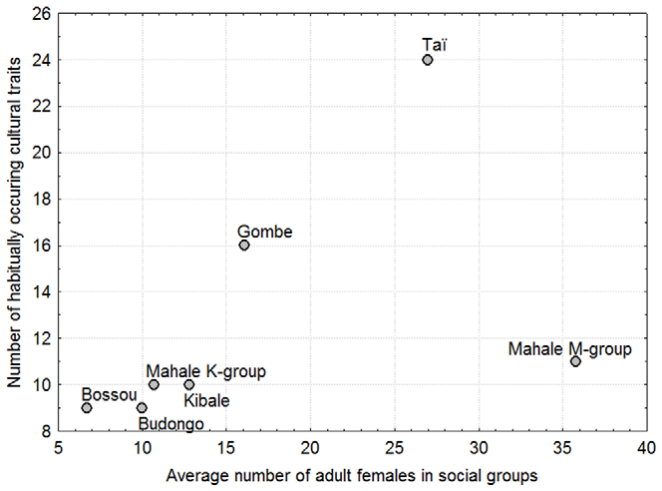
The relationship between female group size and the number of cultural traits reported for seven different chimpanzee communities.

We found a significant correlation between the number of females in chimpanzee communities and the reported number of cultural traits (r_s_ = 0.873, p = 0.010, n = 7) (Fig. 1). Interestingly, we found no such correlation between reported number of cultural traits and male group size (r_s_ = 0.018, p = 0.969, n = 7), and accordingly only an indication of a correlation with total community size (r_s_ = 0.727, p = 0.064, n = 7).

## Discussion

The correlation between female group size and the reported number of cultural traits indicates that chimpanzee cultural carrying capacity depends on the number of females in chimpanzee communities. This implies that females are critical in chimpanzees for transmitting cultural traits and maintaining cultural diversity. The reported pattern may be explained by the fact that females transfer between communities, bringing with them novel cultural traits and consequently increasing the cultural diversity of the community as a whole.

Vast differences exist between the community sizes of humans living in modern societies and chimpanzees, so it may be tempting to infer that the difference in cultural evolution between humans and chimpanzees depends on differences in community size. However, early hominids lived at much lower population densities than contemporary humans and still, as inferred from the archaeological record [14], exhibited more culture than chimpanzees [7]. For example, the presence and diversity of early Oldowan stone tools, dating as far back as more than 2 000 000 years, imply a far more complex and diverse culture than what is observed in chimpanzees [15]. The difference between humans and chimpanzees therefore most probably depends on other traits rather than demography.

In humans, culture can grow exponentially as innovation rates depend on the number of cultural traits already present [16]. The lack of a similar exponential growth of chimpanzee culture (inferred from the fact that they do not possess a large amount of culture at present^1^) might reflect that chimpanzees do not have the mental capacity necessary for making use of established cultural traits when innovating novel traits. This merits further studies of the underlying processes of chimpanzee cultural evolution [14], [16]–[19].

As chimpanzee communities continue to dwindle in Africa [20], more diversity is at stake than biodiversity. If ever lower numbers of chimpanzees results in the transmission of a reduced number of cultural traits over generations and between communities, we risk losing an important possibility of understanding cultural evolution in our closest living relative.

## Materials and Methods

We used data on culture [1] and community size [21] from seven communities of wild chimpanzees from different localities. To avoid bias, we only used behaviour patterns that occurred habitually or customarily in these communities [3]. We also excluded behaviours that are non-informative; either because they occur in all locations or their absence can be explained by local ecological conditions. Hence, for this analysis we have used all informative data available thereby avoiding ambiguities. For full details and explanations see table 1 in Whiten *et al.* 1999^1^. Two estimates of group composition were available for the Gombe site [21], whereof we chose the newer as this more closely matched the cultural data.

We used a phylogeny of chimpanzee communities of Lycett et al. [4] to analyse whether the number of cultural traits and average community sizes showed evidence of phylogenetic signal [13], [22]. Note that this phylogeny was made using the same cultural data that we use in our analyses. Thus, by utilizing this phylogeny we maximally slanted the tests in favour of finding a phylogenetic signal. To test for the presence of a phylogenetic signal, we estimated λ using maximum likelihood [13] in the package APE [23] in the statistical software R [24]. Non-parametric Spearman rank correlations were used for all non-phylogenetic tests because of the unequal distribution of the data, and all p-values are two-tailed.

## References

[1] Whiten A, Goodall J, McGrew WC, Nishida T, Reynolds V (1999). Cultures in chimpanzees.. Nature.

[2] Whiten A, Goodall J, McGrew WC, Nishida T, Reynolds V (2001). Charting cultural variation in chimpanzees.. Behaviour.

[3] van Schaik CP, Ancrenaz M, Borgen G, Galdikas B, Knott CD (2003). Orangutan cultures and the evolution of material culture.. Science.

[4] Lycett SJ, Collard M, McGrew WC (2007). Phylogenetic analyses of behavior support existence of culture among wild chimpanzees.. Proc Natl Acad Sci USA.

[5] Lycett SJ, Collard M, McGrew WC (2009). Cladistic analyses of behavioural variation in wild *Pan troglodytes*: exploring the chimpanzee culture hypothesis.. J Hum Evol.

[6] Strimling P, Sjöstrand J, Enquist M, Eriksson K (2009). Accumulation of independent cultural traits.. Theor Popul Biol.

[7] Powell A, Shennan S, Thomas MG (2009). Late Pleistocene demography and the appearance of modern human behavior.. Science.

[8] Boesch C, Boesch H (1990). Tool use and tool making in wild chimpanzees.. Folia Primatol.

[9] Nishida T, Hiraiwa-Hasegawa M, Smuts BB, Cheney DL, Seyfarth RM, Wrangham RW, Struhsaker TT (1987). Chimpanzees and bonobos: cooperative relationships among males.. Primate Societies.

[10] Hannah AC, McGrew WC (1987). Chimpanzees using stones to crack open oil palm nuts in Liberia.. Primates.

[11] McGrew WC, Hamburg DA, McCown ER (1979). Evolutionary implications of sex differences in chimpanzee predation and tool use..

[12] Lonsdorf EV 2006 What is the role of mothers in the acquisition of termite-fishing behaviors in wild chimpanzees (*Pan troglodytes schweinfurthii*)?. Anim Cogn.

[13] Freckleton RP, Harvey PH, Pagel M (2002). Phylogenetic dependence and ecological data: a test and review of evidence.. Am Nat.

[14] McBrearty S, Brooks A (2000). The revolution that wasn't: a new interpretation of the origin of modern human behavior.. J Hum Evol.

[15] Domínguez-Rodrigo M, Rayne Pickering T, Semaw S, Rogers MJ (2005). Cutmarked bones from Pliocene archaeological sites at Gona, Afar, Ethiopia: implications for the function of the world's oldest stone tools.. J Hum Evol.

[16] Enquist M, Ghirlanda S, Jarrick A, Wachtmeister C-A (2008). Why does human culture increase exponentially?. Theor Popul Biol.

[17] Hrubesch C, Preuschoft S, van Schaik C (2008). Skill mastery inhibits adoption of observed alternative solutions among chimpanzees (*Pan troglodytes*).. Anim Cogn.

[18] Marshall-Pescini S, Whiten A (2008). Chimpanzees (*Pan troglodytes*) and the question of cumulative culture: an experimental approach.. Anim Cogn.

[19] Whiten A, McGuigan N, Marhsall-Pescini S, Hopper LM (2009). Emulation, imitation, overimitation and the scope of culture for child and chimpanzee.. Phil Trans R Soc.

[20] IUCN 2009 IUCN Red List of Threatened Species..

[21] Wrangham RW, Kappeler PM (2000). Why are male chimpanzees more gregarious than mothers? A scramble competition hypothesis.. Primate Males: Causes and Consequences of Variation in Group Composition.

[22] Pagel M (1999). Inferring the historical patterns of biological evolution.. Nature.

[23] Paradis E, Claude J, Strimmer K (2004). APE: analyses of phylogenetics and evolution in R language.. Bioinformatics.

[24] R Development Core Team (2008). R: A language and environment for statistical computing..

